# Radiation Therapy After Radical Prostatectomy: What Has Changed Over Time?

**DOI:** 10.3389/fsurg.2021.691473

**Published:** 2021-07-09

**Authors:** Fabio Zattoni, Isabel Heidegger, Veeru Kasivisvanathan, Alexander Kretschmer, Giancarlo Marra, Alessandro Magli, Felix Preisser, Derya Tilki, Igor Tsaur, Massimo Valerio, Roderick van den Bergh, Claudia Kesch, Francesco Ceci, Christian Fankhauser, Giorgio Gandaglia

**Affiliations:** ^1^Urology Unit, Azienda Sanitaria Universitaria Integrata di Udine, Udine, Italy; ^2^Department of Urology, Medical University Innsbruck, Innsbruck, Austria; ^3^Division of Surgery and Interventional Science, University College London, London, United Kingdom; ^4^Department of Urology, University College London Hospital, London, United Kingdom; ^5^Department of Urology, Ludwig-Maximilians-University of Munich, Munich, Germany; ^6^Department of Urology, San Giovanni Battista Hospital, University of Turin, Turin, Italy; ^7^Department of Radiation Oncology, Udine General Hospital, Udine, Italy; ^8^Department of Urology, University Hospital Frankfurt, Frankfurt, Germany; ^9^Martini-Klinik Prostate Cancer Center, University Hospital Hamburg-Eppendorf, Hamburg, Germany; ^10^Department of Urology, University Hospital Hamburg-Eppendorf, Hamburg, Germany; ^11^Department of Urology and Pediatric Urology, Mainz University Medicine, Mainz, Germany; ^12^Department of Urology, CHUV Lausanne, Lausanne, Switzerland; ^13^Department of Urology, Antonius Hospital, Utrecht, Netherlands; ^14^Department of Urology, University Hospital Essen, Essen, Germany; ^15^Division of Nuclear Medicine, IEO European Institute of Oncology IRCCS, Milan, Italy; ^16^University Hospital Zürich, Zurich, Switzerland; ^17^Division of Oncology/Unit of Urology, Urological Research Institute, IRCCS Ospedale San Raffaele, Milan, Italy

**Keywords:** prostate cancer, adjuvant radiotherapy, salvage radiotherapy, biochemical recurrence, hormonal therapy, genomic classifiers

## Abstract

The role and timing of radiotherapy (RT) in prostate cancer (PCa) patients treated with radical prostatectomy (RP) remains controversial. While recent trials support the oncological safety of early salvage RT (SRT) compared to adjuvant RT (ART) in selected patients, previous randomized studies demonstrated that ART might improve recurrence-free survival in patients at high risk for local recurrence based on adverse pathology. Although ART might improve survival, this approach is characterized by a risk of overtreatment in up to 40% of cases. SRT is defined as the administration of RT to the prostatic bed and to the surrounding tissues in the patient with PSA recurrence after surgery but no evidence of distant metastatic disease. The delivery of salvage therapies exclusively in men who experience biochemical recurrence (BCR) has the potential advantage of reducing the risk of side effects without theoretically compromising outcomes. However, how to select patients at risk of progression who are more likely to benefit from a more aggressive treatment after RP, the exact timing of RT after RP, and the use of hormone therapy and its duration at the time of RT are still open issues. Moreover, what the role of novel imaging techniques and genomic classifiers are in identifying the most optimal post-operative management of PCa patients treated with RP is yet to be clarified. This narrative review summarizes most relevant published data to guide a multidisciplinary team in selecting appropriate candidates for post-prostatectomy radiation therapy.

## Introduction

The most common primary treatment for localized prostate cancer (PCa) is radical prostatectomy (RP) (1). Approximately one third of men managed with RP will experience biochemical recurrence (BCR) over a 10-year period (2), and the majority of these patients will eventually develop distant metastases and/or will die of PCa over time if left untreated (3). Postoperative radiotherapy (RT) represents an option in a multimodal setting in order to reduce the risk of experiencing distant metastases at follow-up. Of note, RT might be administered in an adjuvant (i.e., immediately after surgery in the absence of signs of recurrence) or salvage setting (i.e., at the time of biochemical recurrence, BCR). However, there has been poor consensus regarding the timing of post-operative RT. Previous prospective, randomized clinical trials showed that ART was associated with a reduced risk of recurrence in patients at risk (i.e., positive surgical margins, pT3 disease, pathologic grade group 4–5). However, their generalizability is limited by either late use of SRT or no use of post-RP prostate-specific antigen (PSA) monitoring or both (4–7). More recent randomized studies compared ART with early SRT for patients with an increasing PSA level after RP (early SRT) and provide data which might be applied to contemporary patients (8–10). However, how to select patient at risk of progression who more likely will benefit from a more aggressive treatment after RP in a multimodal setting, the exact timing of RT after RP, and the use of hormone therapy and its duration at the time of RT are still open issues. This is particularly true when considering the poor sensitivity of imaging techniques (transrectal US, CT, pelvic MRI, PET/CT, and PET/MRI with different radiopharmaceuticals) in asymptomatic patients with early BCR after RP. Moreover, molecular biomarkers in this setting have been poorly addressed so far and their use in the clinical practice is still limited (11).

This narrative review summarizes most relevant published data to guide a multidisciplinary team in selecting appropriate candidates for post-prostatectomy radiation therapy after the availably of new landmarks randomized studies.

## Evidence Acquisition

A collaborative non-systematic literature review identified recently published randomized and non-randomized studies where outcome data were collected (cut-off date February 6th 2021). The medical electronic data base PubMed was used. The identified studies represented the basis for a narrative review of the literature analyzing role of ART and SRT for BCR/PSA persistence (BCP) after RP.

## Evidence Synthesis

### Defining Patients at Risk After Radical Prostatectomy

Accurate risk characterization could result in an appropriate management of post-RP patients. However, the optimal post-operative approach to these patients is a subject of continuous debate because the risk definition after RP relies on clinical, pathological features and PSA kinetics. Furthermore, the choice of treatment (initial observation, ART, and/or ADT) should be tailored according to prognostic factors and/or risk stratification.

Up to one-third of patients treated with RP may have adverse pathologic features (12), defined as positive surgical margins, extra-prostatic extension, seminal vesicle invasion, and/or lymph node invasion and high Gleason score.

Only patients with at least two of the following pathologic features are at higher risk of cancer specific mortality and may significantly benefit from adjuvant treatment after RP: pathologic Gleason score ≥8, pT3/pT4 disease, and the presence of nodal disease (≥1) (13).

In the study of Abdollah et al. men with low-volume nodal disease (<3 LNs), ISUP grade 2–5 and pT3–4 or R1, as well as men with 3 to 4 positive nodes were more likely to benefit from RT after surgery, while the other subgroups did not (14).

However, the level of evidence for the management of pN1 patients is still low (15).

The most sensitive and the only validated biomarker for disease persistence and recurrence remains PSA and PSA-based parameters (PSA doubling time and interval to PSA failure). Persistent PSA is defined in the majority of studies as detectable post-RP PSA of ≥0.1 ng/mL within 4 to 8 weeks of surgery and occurs in 5–20% of men after RP (16, 17).

It is likely the expression of persistent local disease or pre-existing metastases and reflect in worse outcomes when compared to men experiencing BCR (18). In highly selected patients with favorable pathologic characteristics PSA persistence might also indicate the presence of benign tissue left *in situ* during the procedure (19). On the other hand, persistent PSA represents one of the worst prognostic factors for risk of metastasis and death (18, 20) when associated with adverse pathologic features (21). In these patients, the use of SRT may improve survival, although available data from number of study does not allow yet to make any clear treatment decision (20, 22).

When considering BCR after RP, the threshold that best predicts further metastases is a PSA level of >0.4 ng/mL and rising (4). However, this value should not be considered as the best cut-off to start further treatments. With access to ultrasensitive PSA testing, a rising PSA level below this level might be a cause for concern. So far, several studies report different cutoffs for defining BCR after RP. Currently the most common BCR definition in studies and guidelines is based on two consecutive PSA values ≥0.2 ng/mL and rising, representing a more sensitive threshold to PSA progression. However, a first rise in PSA levels should not be used as the only landmark to start treatments. Although better oncologic outcomes were noticed when salvage treatment was delivered at lower PSA levels, the accurate timing of its administration depends on pathologic features, functional status, quality of life effects and patient's preferences (23–25). Based on the idea that the patient group experiencing BCR is a heterogeneous group, the EAU guidelines suggested a new stratification which accounts for the factors previously described (excluded PSA persistence). This allows to stratify patients in two risk groups: the EAU low-risk BCR (PSA-DT >1 yr and pathological ISUP grade <4) and EAU high-risk BCR (PSA-DT <1 yr or pathological ISUP grade 4–5) group (26). This novel BCR risk categories could be easily implemented in daily practice and could be precious in the decision-making for post-operative RT.

#### Timing of Radiotherapy After Radical Prostatectomy

**Figure 1:**
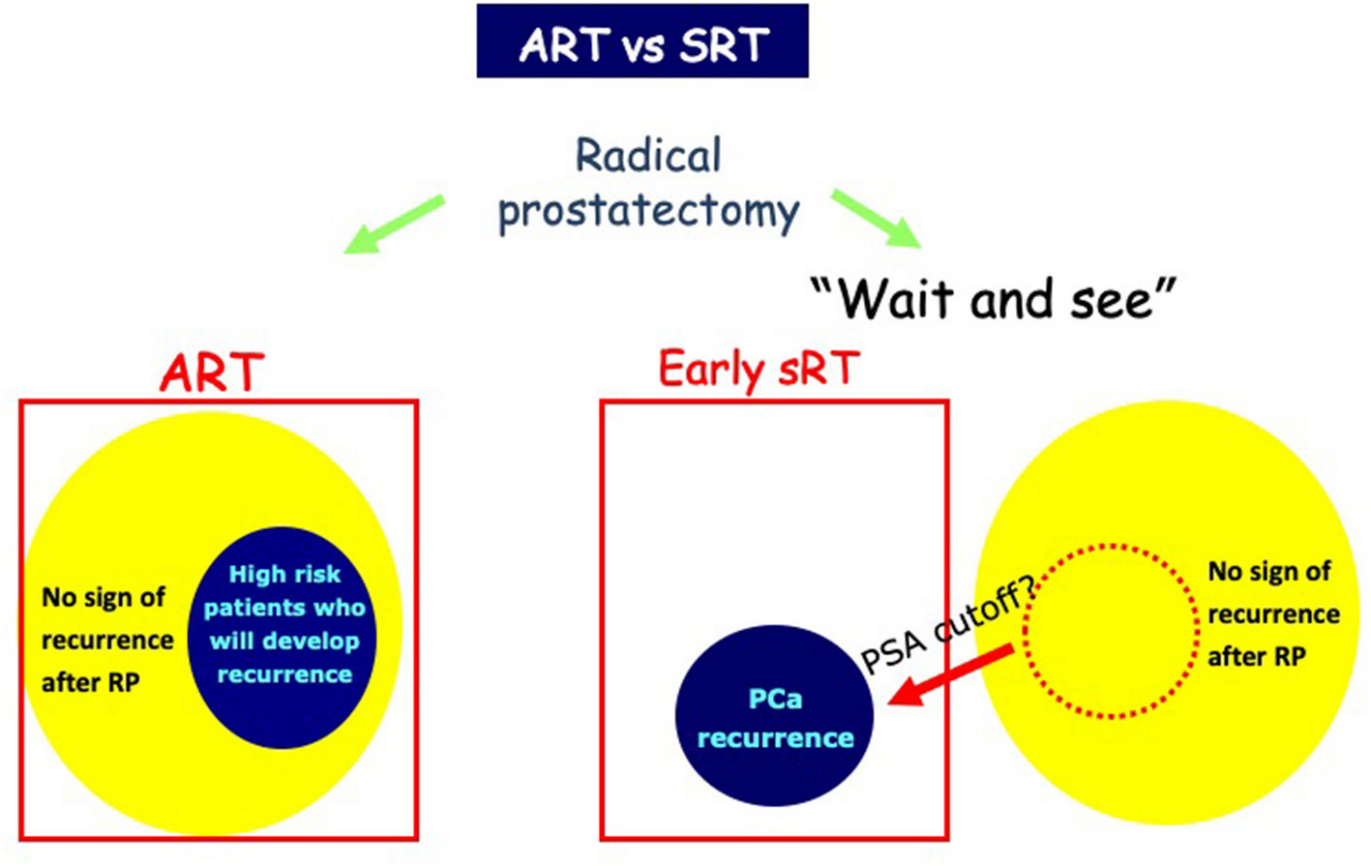
Use of adjuvant RT (ART) and salvage radiotherapy (SRT). Early treatment with ART might be more effective than SRT for biochemical progression. SRT avoids unnecessary treatment of those cured by surgery alone and results in less treatment-related morbidity.

The optimal timing of RT after RP is still debatable (27). Adjuvant treatment has the aim of decreasing the risk of relapse in men without evidence of disease persistence or recurrence after primary treatment when adverse pathologic features are present. On the contrary, SRT consists of the administration of additional therapies at the time of recurrence and represent a curative approach in men experiencing BCR or PSA persistence. The supporters of ART consider the prompt treatment to be more efficient with reduced risk of BCR and clinical recurrence, with acceptable toxicity. On the other hand, SRT may reduce exposure to unnecessary risks and toxicity (Figure 1). In addition, the impact of ART on survival remains controversial.

Seven randomized controlled trials have assessed the outcomes of ART after RP. These trials can be subdivided into two groups: (1) older trials such as the SWOG 8794 (5), EORTC 22911 (4), ARO 96-02 (6) and the FinnProstate Group trial (7) where timely SRT was not always used in the control arm; (2) modern trials such as RADICALS-RT (8), RAVES (9) and GETUG-17 (10) which mandated early SRT for PSA failure in the control arm (Table 1). Randomized trials testing the role of ART [SWOG 8794 (5), EORTC 22911(4), ARO 96–02 (6), FinnProstate Group trial (7)] provided level I evidence regarding the improvement of biochemical control (bPFS), however with no clear advantage in terms of metastasis-free survival (MFS) and overall survival (OS). A recent metanalysis of published randomized trials evaluating ART detected a significant improvement over a 10-year period in clinical progression and presentation of metastases, especially in patients with positive margins (28). However, there is no evidence of improved OS. The toxicity deriving from immediate radiotherapy proved acceptable with only mild increase of genitourinary toxicity (urethral stenosis and urinary incontinence) and rectal toxicity (28). However, it should be emphasized that none of the above-mentioned studies was conducted to confront ART and SRT, the studies had small sample size cohorts for OS analysis and ~30% of the enrolled patients in the SWOG and EORTC trial have received SRT after initial radiotherapy and PSA persistence. On the contrary, there is an evidence that approximately 50% of patients enrolled in these studies did not experience BCR. Thus, the administration of ART in up to half of patients with adverse pathologic characteristics at RP would represent an overtreatment and would expose patients to treatment-related side effects without oncologic benefits.

**Table 1:** Summary of recently published randomized trials for ART.

	**Radicals-RT**	**GETUG-AFU 17**	**Raves**
**Trial design**	Superiority	Superiority	Non-inferiority
**Patients randomized**	Adjuvant: 697 Early salvage: 699	Adjuvant:212 Early salvage: 212	Adjuvant: 166 Early salvage: 167
**Key eligibility criteria**	One or more of: - Positive margins - pT3a, pT3b, or pT4 - or Gleason 7–10	- pT3a, pT3b, or pT4a (with bladder neck invasion); - Positive margins; - Extracapsular extension	- pT2, pT3a, or pT3b AND - Either positive margins - Or extracapsular extension
**Trigger for early salvage radiotherapy**	PSA >0.1 ng/mL and rising or three consecutive rising PSA levels still below 0.1 ng/mL	PSA ≥ 0.20 ng/mL and rising	PSA ≥ 0.20 ng/mL
**Early salvage radiotherapy timing**	≤ 2 months of trigger PSA	As soon as possible after PSA relapse and before PSA of 1 ng/mL	≤ 4 months of trigger PSA
**Adjuvant radiotherapy timing**	≤ 6 months of radical prostatectomy ≤ 2	≤ 6 months of radical prostatectomy As	≤ 6 months of radical prostatectomy ≤ 4
**Use of hormone therapy**	Participants could choose to enter a second randomisation to no hormones or hormones for 6 or 24 months' duration; participants not randomized could receive hormone therapy off protocol	Yes, all patients	No
**Primary endpoint**	Freedom from distant metastases	Event-free survival	Freedom from biochemical progression
**Urinary incontinence**	Self-reported urinary incontinence was worse at 1 year for those in the adjuvant radiotherapy group (mean score 4.8 vs. 4.0; *p* = 0.0023)	Adjuvant: 116/212 (55%) Early salvage: 35/212 (17%)	N/A
**Urinary disorder**	Urethral stricture: Grade 3–4 within 2 years in 6% in the adjuvant radiotherapy group vs. 4% in the salvage radiotherapy group (*p* = 0.020)	- Urinary retention: Adjuvant: 6/212 (3%) Early salvage: 5/212 (2%) - Micturition disorder Adjuvant: 2/212 (1%) Early salvage: 0	≥grade 2 genitourinary toxicity rate (CTCAE*) Salvage radiotherapy (90/167 (54%) Adjuvant (116/166 (70%) OR mixed 0.34, (95% CI 0.17–0.68; *p* = 0.0022)

CTCAE^*^, Adverse events were scored by clinicians per National Cancer Institute Common Terminology Criteria for Adverse Events (CTCAE), version 3.0.14. The CTCAE genitourinary domains included cystitis, urinary incontinence, urethral stricture or stenosis, urinary frequency or urgency, urinary retention, and haemorrhage (genitourinary). Gastrointestinal domains included diarrhoea, proctitis, haemorrhage (rectal), and incontinence (anal).

The FinnProstate Group trial (7) was conducted using higher radiation dose, modern technique and adequate follow-up on one hand, but on the other hand the study had a small sample size with about 50% of patients enrolled in both arms of the trial who had initial PSA <0.2 ng/ml. The trial included patients with pT2 positive surgical margins or pT3a (no pT3b) and showed that 40% of the patients developed biochemical progression. The main advantage of ART in terms of BCR was observed in patients with pT2 or positive surgical margins. Most patients who did not receive ART developed metastatic disease; ART was associated with negligible genitourinary toxicity. The most interesting fact is that patients with BCR who did not undergo ART received SRT at a median PSA of 0.7 ng/ml (late SRT) and 75% of these patients had no evidence of disease at last follow-up. This might confirm a certain effectiveness of late SRT in patients with low-risk factors.

The probability of success of SRT is conditioned by several risk factors for disease progression: pre-SRT PSA values, GS>7, seminal vesicles invasion, PSA-DT <10–12 months, and negative surgical margins. As for PSA values, an increase of 0.1 ng/ml is followed by a loss of 2.6% of bPFS, with a level 2a evidence for initiating SRT at the lowest possible PSA (29). The authors of the study also suggest that a rising post-operative PSA > 0.05 ng/mL might be a reliable indicator of biochemical failure, which justifies the initiation of SRT before PSA reaches a level of >0.2 ng/mL. A very early administration of SRT (PSA <0.2 ng/ml) seem to be more efficient than the early SRT (eSRT) (0.2 ng/ml < PSA <0.5 ng/ml) or late SRT (PSA <0.5-1 ng/ml), particularly in presence of multiple risk factors (pT3b-T4, negative surgical margins, GS>7) (23). All studies that retrospectively confronted ART vs. SRT, showing benefit of ART, present several biases, such as “lead-time bias,” difficult to remove even with sophisticated statistical techniques. Another limitation of the studies, both randomized and non-randomized, is that they refer to data gathered in an era where conventional imaging was not able to assess the presence of disease. Furthermore, there are other points that need to be clarified in order to optimize the use of post-operative RT: total radiation dose, pelvic lymph-node irradiation, combination with hormone therapy.

The three more recent randomized trials (RADICALS-RT, GETUG-AFU 17, and RAVES) evaluated the optimal timing between surgery and start of post-operative RT. Despite some differences such as patient selection, trigger PSA levels for SRT (PSA 0.1 ng/ml in RADICALS; PSA 0.2 ng/ml for other two studies), study design and primary endpoint, their objective was to compare ART and eSRT. RADICALS-RT (8) randomly assigned 1,396 patients at risk for progression to ART or SRT for PSA progression. The primary outcome of the study was freedom from distant metastases. The RADICALS-RT authors reported 5-year biochemical progression-free survival of 85% for patients in the ART group and 88% for those in the SRT group [hazard ratio (HR) 1.10, 95% CI 0.81–1.49; *p* = 0.56] after a median follow-up of 4.9 years. Thus, the authors concluded that an observation policy with PSA controls and SRT in case of PSA progression should be the standard of care after RP. However, this study might be underpowered for patients with a high risk for progression, and a potential benefit of ART may be underestimated by including many patients with favorable risk disease. Interestingly, the presence of lymph node invasion at final pathology represented an exclusion criterion (8). GETUG-AFU 1710 (10) randomized trial aimed to compare ART vs. eSRT after RP combined with short-term ADT in nearly all men. The results of the study suggest that there is no benefit for event-free survival in patients assigned to ART compared with patients assigned to SRT. However, ART can delay time to progression and fewer men had undergone SRT compared with ART. The RAVES study (9) was designed to assess whether freedom from biochemical progression with SRT was non-inferior to ART in patients with extra-prostatic extension, seminal vesicle invasion, or positive surgical margins. HRs favoring SRT in the high-risk subgroups including seminal vesicle invasion and Gleason score of 8–10 in the RAVES study can be explained by a later time observation of PSA progression in the SRT group than in the ART group.

The ARTISTIC collaborative meta-analysis and systematic review (30) was prospectively designed before the results from the three randomized clinical trials were known. It included 2,153 men from the three recent randomized trials and showed no evidence that event-free survival, which was driven by PSA progression, was improved with use of ART compared to SRT in men with localized or locally advanced PCa. Unfortunately, a final recommendation for the use of ART or SRT cannot be made yet. Several limitations of the available literature regarding the use of RT after RP, including lack of group uniformity in pathological risk factors; variability in PSA assay sensitivity and failure criteria; heterogeneity of RT dose and techniques; lack of studies with long follow-up duration; and the use of BCR as an outcome surrogate. Less information was available regarding metastatic recurrence, cancer-specific survival, and overall survival. The patient eligibility criteria for RADICALS-RT included patients who would not receive ART in typical clinical practice because of the low risk of recurrence. Observation of PSA progression in the salvage radiotherapy group occurs at a later time than in the adjuvant radiotherapy group, which can explain a better survivorship favoring SRT in the RADICALS-RT study and in the high-risk subgroups including seminal vesicle invasion and Gleason score ≥8 in the RAVES study. Finally, androgen-deprivation therapy (ADT) can delay time to progression and fewer men had undergone salvage compared with adjuvant radiotherapy in the RADICALS-RT and GETUG-AFU 17 trials—concurrent androgen-deprivation therapy with radiotherapy was used in some men in RADICALS-RT and nearly all men in GETUG-AFU 17 (27).

Postoperative RT may have a detrimental effect on functional outcomes, such as urinary continence and erectile function (31, 32). As such, the identification of the appropriate timing to initiate early SRT is of utmost importance to maximize cancer control and to avoid overtreatment. Recovery from urinary incontinence after RP occurs at a lower rate in patients after ART compared with SRT (31, 33). Concordant data from recent randomized studies showed worse late urinary incontinence or grade ≥3 urinary complications in patients in the SRT group (Table 1).

**Figure 2:**
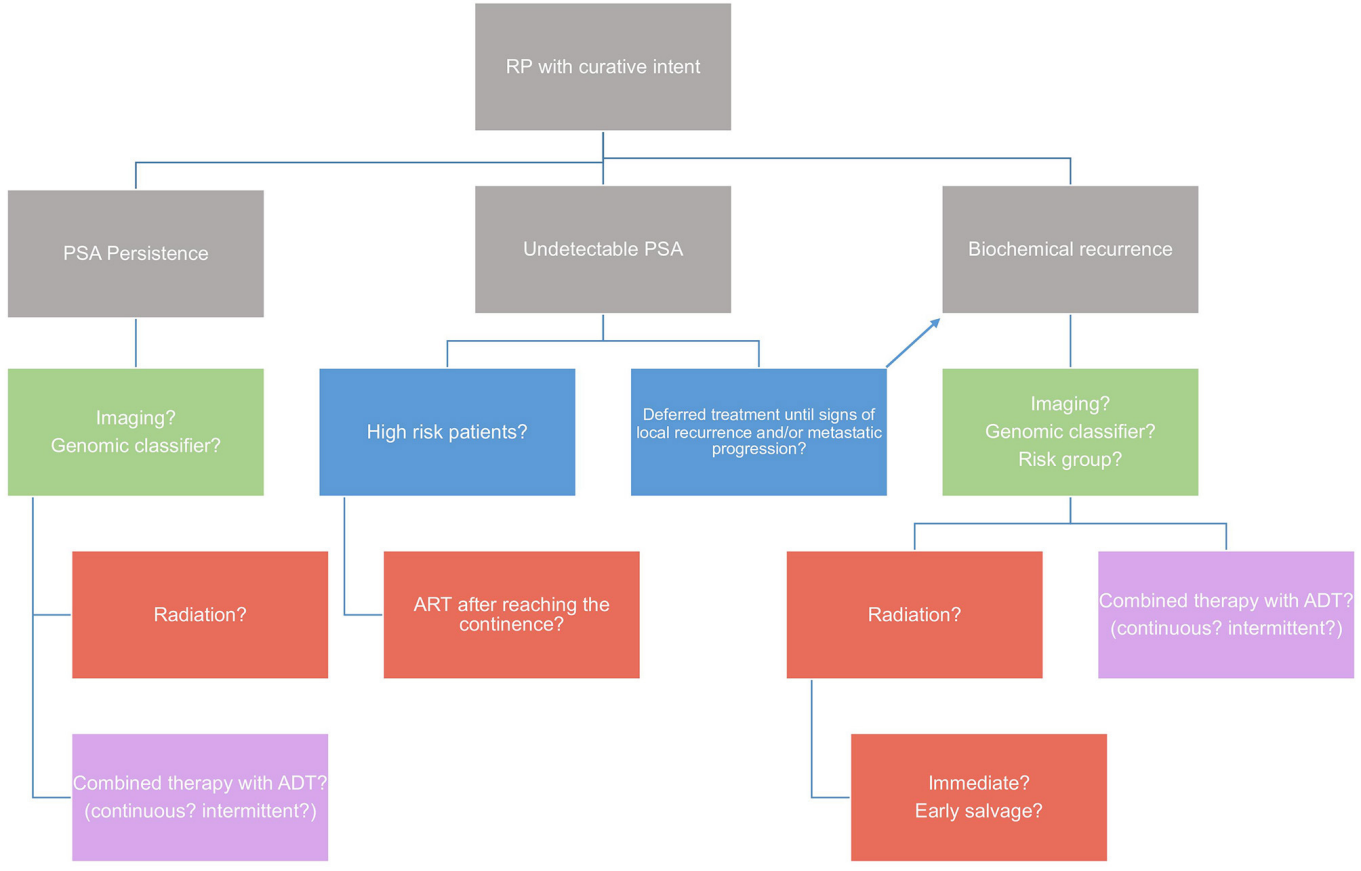
Algorithm on treatment recommendations for the use of ART and SRT after radical prostatectomy. A final recommendation cannot be made yet because several questions are still open.

An algorithm try to summarize the treatment recommendations for the use of ART and SRT after RP (Figure 2). A final recommendation cannot be made yet because several questions are still open.

#### ADT Plus Radiotherapy

The use of ADT in conjunction with RT in the post-RP patient remains controversial. The main questions are whether, when, for how long and in what form ADT should be administered. Available literature has methodological weaknesses since there is a large difference in ADT protocols including when it was administered (e.g., pre-RP, pre-RT, during RT, post-RT), for how long (e.g., months vs. years), differences in RT techniques, targets, total dose administered and study oncologic outcomes.

There are some observational studies which compare RT with or without some form of hormone therapy or antiandrogenic therapy (34–38). Four studies reported findings suggesting that patients who received ADT in combination with ART had better outcomes (bRFS), however only one study reported a statistically significant difference between the two groups. Specifically, Bastide et al. (34) reported that adjuvant ADT combined with RT after RP in patients with SVI resulted in a substantial benefit in 5 year bRFS.

In the retrospective study by Ost et al. (35) the addition of ADT to high dose ART showed significantly improved bRFS and clinical recurrence-free survival (cRFS). Around 30% of patients in RADICALS-RT reported receiving ADT with their post-operative radiotherapy. Although greater use of ADT might have improved outcomes, there is no evidence that it would have had a differential effect on the two arms of the trial. There are several observational studies evaluating post RP patients who received SRT alone compared to those who received SRT in combination with some type of ADT. Most of these suggest better outcomes for patients selected for SRT in combination with ADT.

Evidence from previous trials suggest that men receiving SRT benefit from the addition of ADT: RTOG 9601 showed an advantage in both, cancer-specific survival (CSS) and OS, for the use of 2 years bicalutamide (for all PSA values and for PSA > 1.5 ng/ml) and GETUG-AFU 16 showed an advantage in progression free survival and metastasis-free survival, for the use of 6 months Goserelin (39, 40). However, the offering of hormone therapy should be accompanied by a thorough discussion of the potential benefits and risks/burdens associated with its use in the SRT setting.

In a retrospective multicenter study including 525 patients reported that only in patients with more aggressive disease characteristics (pT3b/4 and ISUP grade >4, or pT3b/4 and a PSA level at early SRT of >0.4 ng/mL), the administration of concomitant ADT for more that 12 months resulted in a reduction in distant metastases (41). Likewise, in a retrospective study of 1,125 patients, three risk factors (stage ≥pT3b, Gleason score ≥8, and a PSA level at SRT of >5 ng/mL) for clinical recurrence were evaluated to determine which patients may benefit from long-term concomitant hormonal therapy (median ADT duration of 9 months). Their data suggest a significant effect of long-term ADT for patients with two or more adverse features. For patients with a single risk factor, short-term ADT (<12 mo) was slightly beneficial whereas patients without risk factors did not show a benefit from concomitant ADT (42). As a limitation of the study, the indication for concomitant ADT, the type of drug administered, and the treatment duration were left at the discretion of the treating physician on the basis of individual patient characteristics.

#### Imaging and Genetic Testing Before ART/SRT

The decision to offer RT in recurrent PCa can be challenging. A proper patient selection is essential to ensure favorable outcomes. Patients usually undergo SRT without local imaging because SRT is usually delivered because of PSA values (ideally when the PSA level <0.5 ng/mL), without histological conformation of local recurrence. In addition, the dose delivered to the prostatic fossa tends to be uniform since it has not been demonstrated that stereotaxic boost to the recurrence site during SRT improves the oncologic outcome with comparable patient reported genitourinary symptom burden (26, 43).

Modern imaging modalities may provide earlier and accurate identification of sites of recurrences in the pelvic area and thus result in change in RT planning of the irradiation field and improvement in oncological outcomes. In certain cases, PSA levels have limited correlation with tumor burden, and patients with poorly differentiated tumors may have metastatic disease in the absence of significantly elevated PSA levels.

Multiparametric MRI of the pelvis is accurate to correctly identify local recurrence in patients with BCR after RP (44, 45). However, its sensitivity in patients with PSA level <0.5 ng/mL remains controversial (45–47). To promote standardization and reduce variations in the acquisition, interpretation, and reporting of local PCa recurrence recently has been proposed a codified method for image acquisition and assessment of PCa local recurrence using MRI after RP (PI-RR) (48). At the moment, whole-body MRI in detecting occult bone or LN metastases in the case of BCR requires further assessment.

After RP, transrectal ultrasound can occasionally show local recurrence as a hypoechoic nodular mass identified in the perianastomotic area. The detection rates in a subgroup of patients with rising PSA ≤ 0.5 ng/ml are ranging between 28.1 and 73.0% (49, 50). The sensitivity however of anastomotic biopsies is low, especially for PSA levels <1 ng/mL (51). The prostatic fossa is notoriously difficult to biopsy and MR-TRUS fusion-guidance may aid in the localization of targets compared to TRUS-guidance alone (52). One implication of accurately localizing recurrences is that it enables targeted boost radiotherapy to confirmed lesions which is thought to improve response (53).

At the moment, prostate-specific membrane antigen PET/CT has shown good potential in patients with BCR, even with PSA levels <0.5 ng/mL (54) with a detection rate around 33–45% (55). Promising results for PET/CT are coming from not only retrospective studies but also from recent prospective trials.

68Ga-PSMA-11 PET accuracy in a prospective multicenter trial have showed 84 to 92% positive predictive value, 75% overall detection rate increasing with PSA values (38% for <0.001, 57% for 0.5 to <1.0 ng/mL, 84% for 1.0 to <2.0 ng/mL, 86% for 2.0 to <5.0 ng/mL, and 97% for 5.0 ng/mL), a good inter-reader reproducibility and safety (56).

According to a systematic review and metanalysis (57), for PSA categories 0–0.19, 0.2–0.49, 0.5–0.99,1–1.99, and >2 ng/ml, the percentages of positive scans are 33, 46, 57, 82, and 97%, respectively.

In OSPREY prospective trial, the diagnostic performance of PSMA PET/CT was assed to determine sites of metastatic PCa. In post-therapy men with suspected recurrent or metastatic disease, PSMA PET/CT demonstrated high sensitivity (>88%) and PPV (≥75%) in all sites of disease and across all PSA ranges (58). The use of a histopathologic biopsy as gold standard for all patients and a blinded, independent reader paradigm is a distinct feature of OSPREY study in establishing diagnostic performance.

In 208 patients with BCR (PSA ranging between 0.2 and 98.4 ng/mL) and negative standard imaging the performance of PSMA PET/CT (CONDOR study) was found to determine a correct localization rate of 84.8–87.0%. Interestingly 63.9% of evaluable patients had a change in intended management after PSMA PET/CT (59).

However, men with recurrent/persistent disease reflect different clinical settings and highly heterogeneous population, carrying different prognosis and different profiles of disease aggressiveness. Therefore, selecting the most suitable candidates for PSMA PET/CT is critical to optimize its use and to spare lower-risk patients by expensive and potentially unnecessary staging procedures. By identifying patients with high probability to result in positive PSMA PET/CT, suspicious PCa recurrence could be identified and treatment strategies adjusted accordingly. Nomogram might represent a comprehensive and useful tool in guiding physicians in the most appropriate use of PSMA PET/CT. Models include pathologic parameters (ISUP grade), biochemical characteristics (PSA, PSAdt, ongoing ADT, and time to relapse) and the clinical settings of PSA relapse. Nomogram may allows a smoother patient selection by the clinician, prior to imaging referral in comparison to the use of the PSA values only (60–62).

Sites of recurrence can be clarified by PSMA PET and disease localization may translate into management changes in >50% of patients with BCR (63). Thus, SRT may represent a future strategy in case of BCR where PSMA PET rules out metastatic disease.

In a recent systematic review and meta-analysis (64), PET/MRI seems to have a pooled detection rate of 80.9% (95% CI 73.0–86.9%). However, heterogeneity among the studies was very high. Interestingly, both Grubmuller et al. (65) and Hope et al. (66) reported a high detection rate for recurrent PCa even at very low PSA levels (<0.5 ng/mL). This may prompt changes in RT planning. It is worth noting that the term “PSMA PET” refers to several different radiopharmaceuticals and at present there are no conclusive data about comparison of such tracers. Little difference in terms of detection rate was revealed between the three most commonly used PSMA-radiotracers (68Ga-PSMA11, 18F-PSMA-1007, 18F-DCFPyl), which in turn showed clear superiority to choline and fluciclovine. In a network meta-analysis, 18F-PSMA-1007 is favored in all pairwise comparisons. However, there is currently insufficient evidence to favor any routinely used PSMA-radioligands over another owing to the limited evidence base and risk of publication bias (67).

For the future, new PET tracers and the extraction and quantification of MRI imaging features (radiomics) (68, 69) may guide future research in patients stratification into high potential responder (negative findings or recurrence confined to the prostate) and poor potential responder (positive nodes or distant disease) to SRT.

Genomic markers have been proposed as a complementary tool for risk stratification in patients with PCa. These markers capture genomic information specific to each patient's tumor which is beyond routinely available clinical and pathologic characteristics (tumor stage, grade, PSA value). In the last decade, there has been heightened interest in exploring the utility of different genomic signatures that serve as prognostic markers of cancer control in patients newly diagnosed with localized PCa as well as in patients who have undergone RP. Several novel biomarkers have been introduced for the diagnostic (PHI®, 4K score, SelectMDx®, ConfirmMDx®, PCA3, MiPS, ExoDX®, mpMRI) and prognostic purpose (OncotypeDX GPS®, Prolaris®, ProMark®, DNA-ploidy, Decipher®) (70).

The most utilized test in the real world practice is Decipher, which has been shown to correlate with increased cumulative incidence of BCR, metastasis and PCa-specific mortality (70, 71).

A recent systematic review (11) evaluated the clinical effectiveness of the Decipher genomic classifier (GC) for men with PCa. The authors found consistent evidence that the test may help to identify which cancers are more or less aggressive.

Decipher GC is prognostic for long-term metastasis/survival and changes management of PCa in the post-RP setting. Results have been demonstrated in prospective and *post-hoc* analysis of randomized clinical trials. Furthermore, GC results predict benefit from receipt of treatment which in turn supports personalized treatment decision-making in post-RP patients.

In this particular setting, Decipher GC may guide ART or SRT after RP based on a discrete cut-off score. Moreover, in patients who have already harbored BCR, it can guide decisions regarding the need for early/multimodal SRT vs. SRT alone. Interestingly, patients with higher Decipher GC scores were found to have more metastatic lymph node involvement on PSMA PET-imaging in a study population with 48% of prostatectomy patients. These suggests that patients with GC high risk might benefit from more nodal imaging and treatment intensification (72).

The Decipher GC met high level evidence in post-prostatectomy setting for both Simon and AUA criteria (11). This said, the evidence supports a routine use in clinical situations that will change patient management.

## Conclusion

The three most recent randomized trials RADICALS-RT, GETUG-AFU 17, and RAVES and the ARTISTIC metanalysis all conclude that SRT may offer the opportunity to avoid, or at least postpone, radiotherapy and its associated side effects for many men with no obvious disadvantage to event-free survival.

However, in daily practice ART should be proposed to patients with PSA persistence, EAU high-risk group or to patients with undetectable PSA values but with multiple high-risk factors (seminal vesicle invasion, GS > 7). Whereas, in patients with undetectable PSA values, EAU low-risk group and no high-risk factors (e.g., pT2/SM + or pT3a/ SM + or GS <8 and nerve sparing surgery) SRT should be considered in cases when PSA levels rise (>0.2 ng/ml).

In the nearer future, molecular biomarkers, clinical and histopathological features and imaging diagnostics will have to be used in a complementary fashion in order to provide the best possible patient selection. Further prospective studies are needed to confirm these conclusions.

## Author Contributions

FZ and GG: conceived the study. FZ and AM: collected the data. FZ, GG, IH, VK, and AK: wrote the manuscript. GM, FP, CK, FC, and CF: interpretation of results. DT, IT, MV, and RB: supervision. All authors reviewed the results and approved the final version of the manuscript.

## Conflict of Interest

The authors declare that the research was conducted in the absence of any commercial or financial relationships that could be construed as a potential conflict of interest. The handling editor declared a shared affiliation with one of the authors FP at the time of review.
